# Supplementary material for: Hydrogen production by Sulfurospirillum species enables syntrophic interactions of Epsilonproteobacteria
Source: Nat Commun. 2018 Nov 19;9:4872. doi: 10.1038/s41467-018-07342-3 (PMC6242987; doi:10.1038/s41467-018-07342-3)
Supplement: Supplementary file 1 — Supplementary Information [file 41467_2018_7342_MOESM1_ESM.pdf]

Hydrogen production by *Sulfurospirillum* species enables syntrophic interactions of Epsilonproteobacteria

Stefan Kruse[1,§], Tobias Goris[1,§,#], Martin Westermann[2], Lorenz Adrian[3,4], Gabriele Diekert[1]

[1]Department of Applied and Ecological Microbiology, Institute of Microbiology, Friedrich Schiller University, 07743 Jena, Germany

[2]Center for Electron Microscopy of the University Hospital Jena, Jena, Germany

[3]Department Isotope Biogeochemistry, Helmholtz Centre for Environmental Research – UFZ, Permoserstr. 15, 04318 Leipzig, Germany

[4]Technische Universität Berlin, Fachgebiet Geobiotechnologie, Ackerstraße 76, 13355 Berlin

Corresponding author: Tobias Goris (E-Mail: tobias.goris@uni-jena.de; tobiasgoris@gmail.com)

Supplementary Figures and Tables

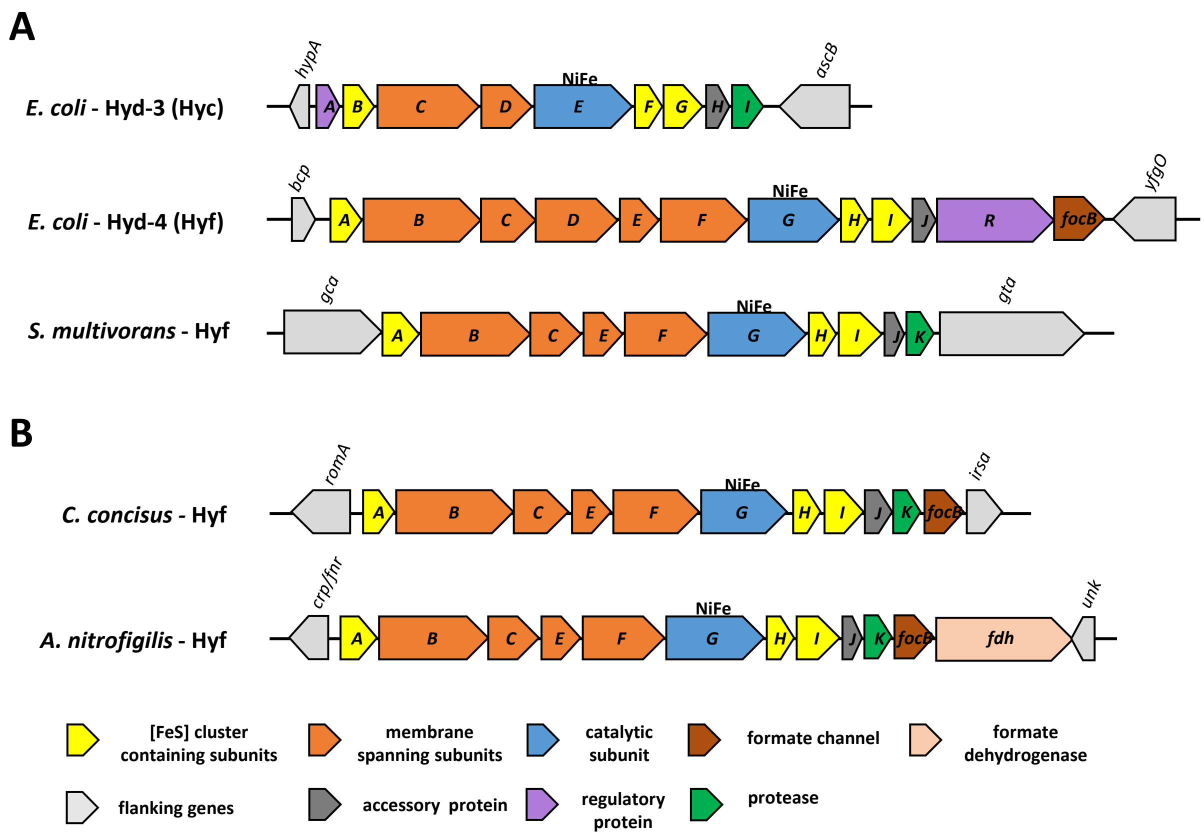

Supplementary Figure 1: Comparison of the *S. multivorans hyf* gene cluster to those encoding Hyd-3 and Hyd-4 of *E. coli* (A) and to other epsilonprotobacterial *hyf* clusters (B). Genetic organization and flanking genes around the operons are depicted. *C. concisus* - *Campylobacter concisus*, *A. nitrofigilis* - *Arcobacter nitrofigilis.* NiFe - catalytic subunit, - *hypA* - NiFe hydrogenase maturation protein, *ascB* - β-glucosidase, *bcp* - putative thiol peroxidase, *yfgO* - putative permease (PerM), *gca* - diguanylate cyclase, *gta* - putative β-glucosyltransferase, *romA* - outer membrane protein, *irsa* - putative membrane protein, *crp/fnr* - putative transcriptional regulator, *unk* - unknown protein.

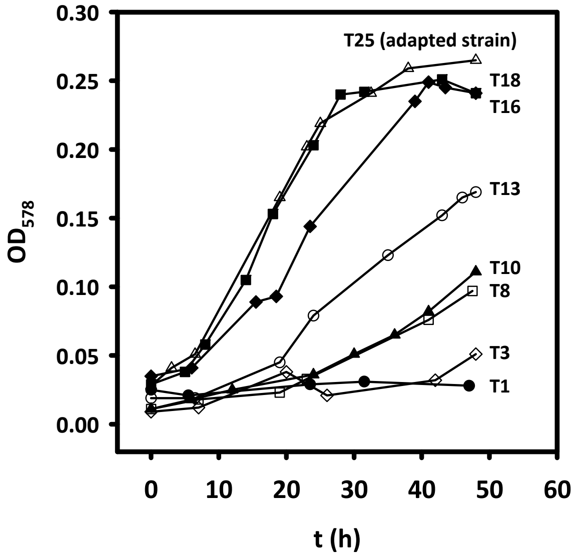

Supplementary Figure 2: Adaptation of *S. multivorans* to pyruvate-fermenting conditions. The graph includes the second biological independent replicate and corresponds to Figure 1B of the main manuscript. T - number of transfer step, OD_{578} - optical density at 578 nm.

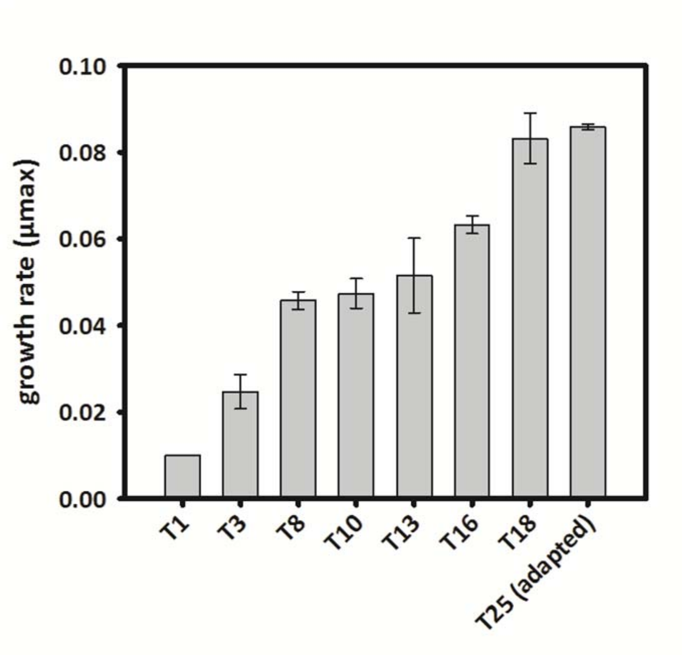

Supplementary Figure 3: Adaptation to pyruvate fermentation of *S. multivorans*. Increase of growth rate during continuous transfer to pyruvate medium without an electron acceptor. Data represent mean of two independent replicates with error bars. T - transfer step.

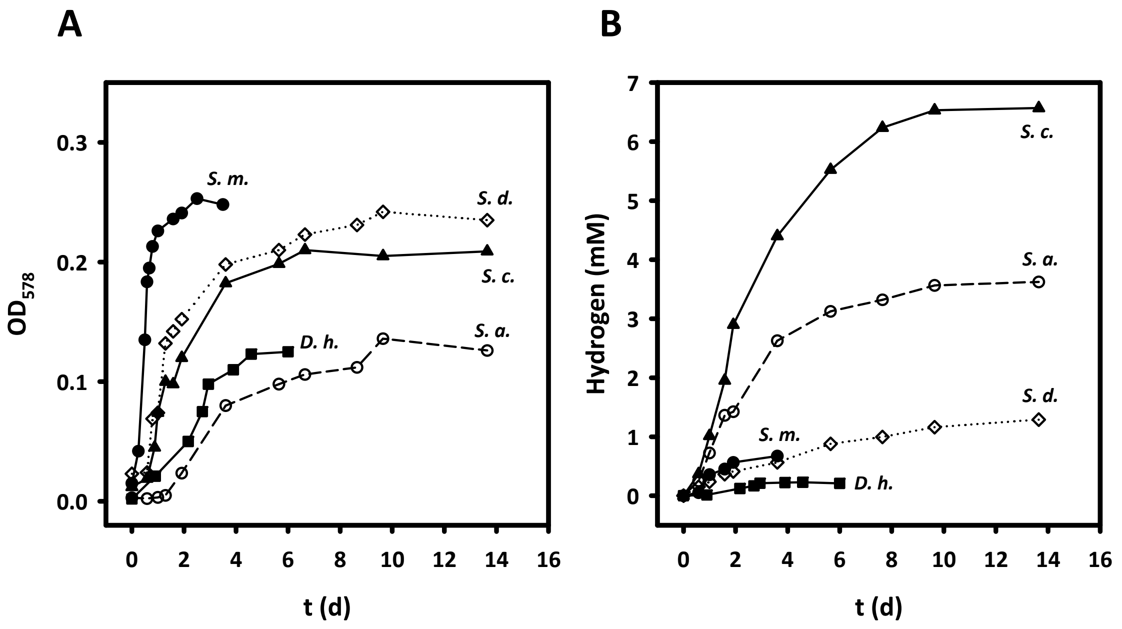

Supplementary Figure 4: Growth and H_2 production during fermentative growth, replicate 2 . The graph shows the second biological independent replicate and corresponds to Figure 2 of the main manuscript. *S.m.* - *S. multivorans*, *S.d.* - *S. deleyianum*, *S.c.* - *S. cavolei*, *S.a.* - *S. arsenophilum*, *D.h.* - *D. hafniense* DCB-2.

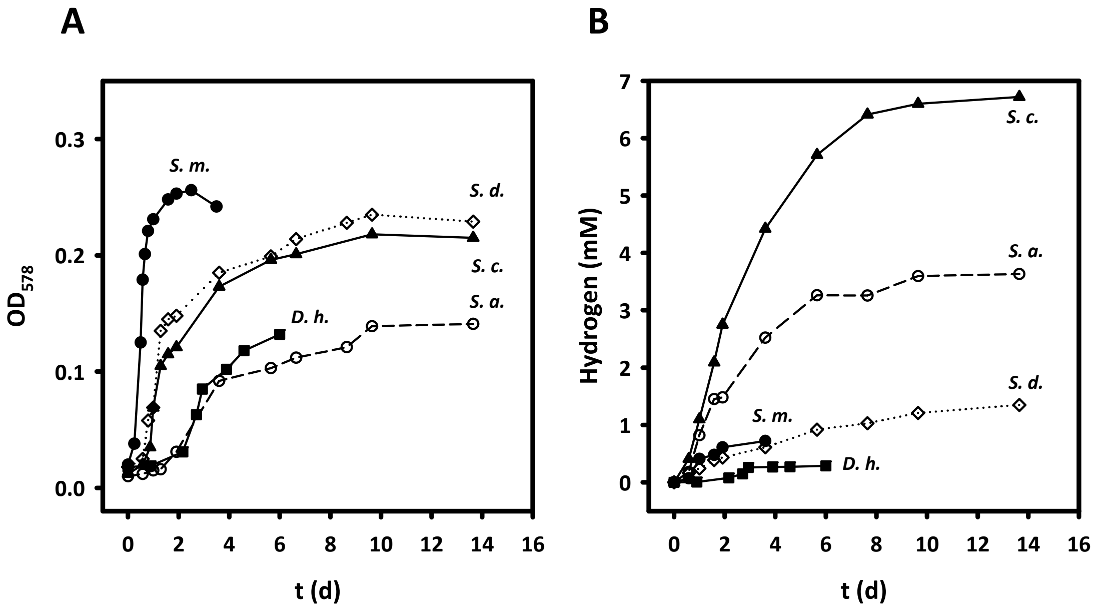

Supplementary Figure 5: Growth and H_2 production during fermentative growth, replicate 3. The graph shows the third biological independent replicate and corresponds to Figure 2 of the main manuscript. *S.m.* - *S. multivorans*, *S.d.* - *S. deleyianum*, *S.c.* - *S. cavolei*, *S.a.* - *S. arsenophilum*, *D.h.* - *D. hafniense* DCB-2.

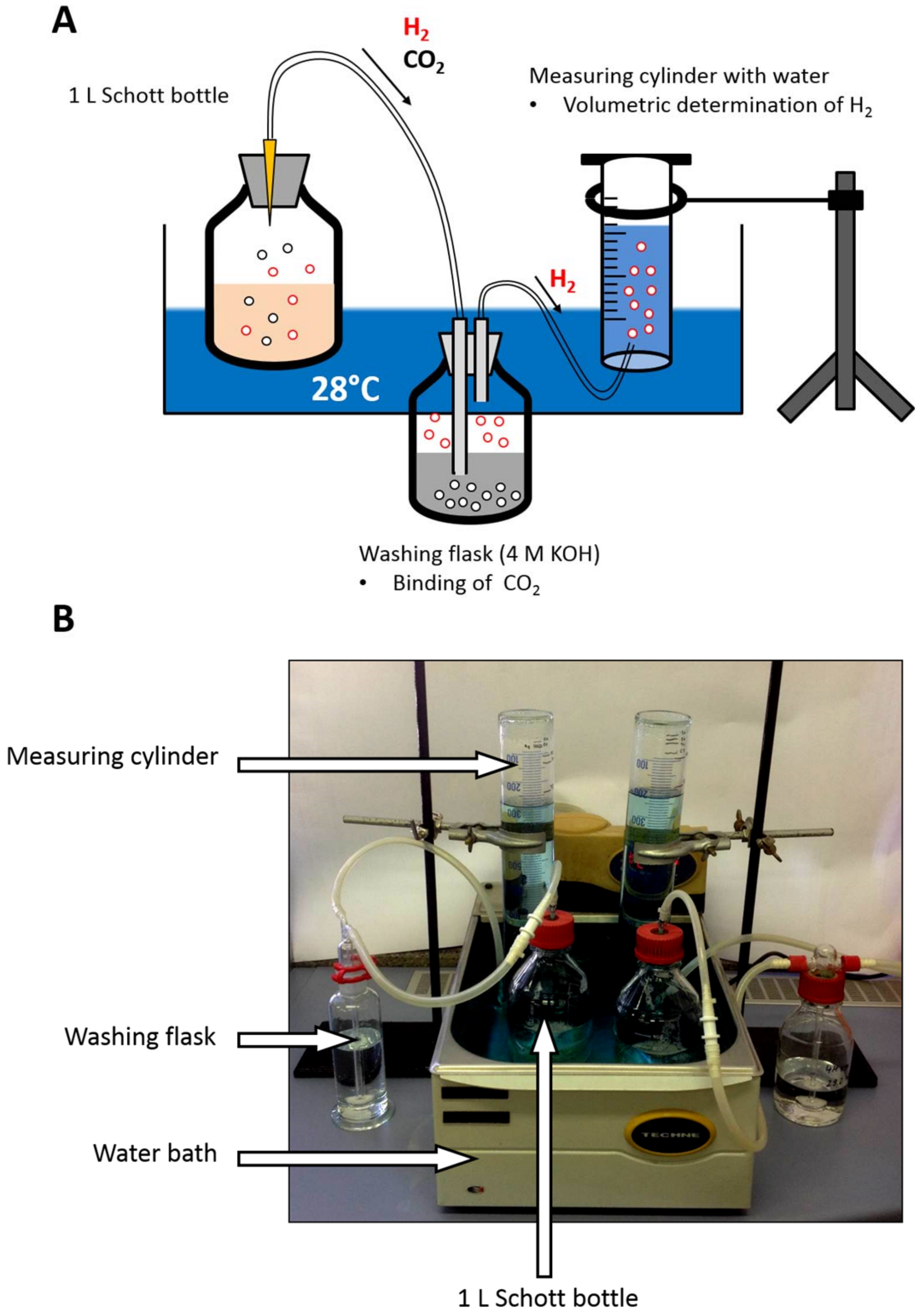

Supplementary Figure 6: Experimental setup of the fermentation apparatus. Scheme (A) and picture of the The washing flask with 4 M KOH is placed outside of the waterbath.

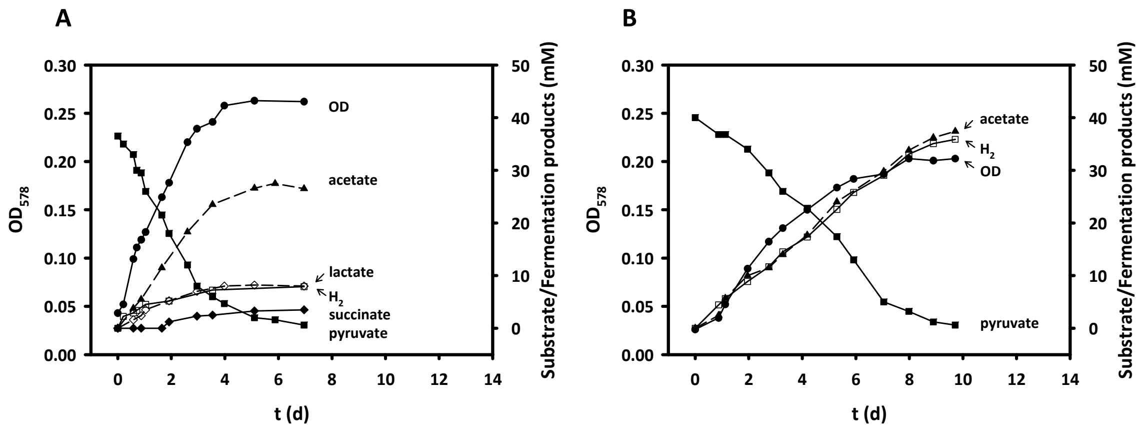

Supplementary Figure 7: Fermentation balance of *S. multivorans* (A) and *S. cavolei* (B) during fermentative growth on 40 mM pyruvate measured in the fermentation apparatus. The graph includes the second biological independent replicate and corresponds to Figure 3 of the main manuscript.

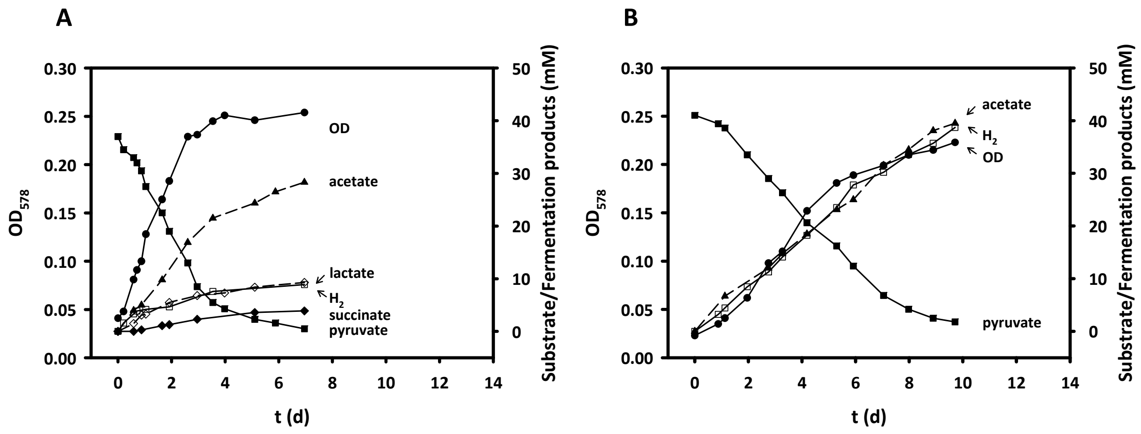

Supplementary Figure 8: Fermentation balance of *S. multivorans* (A) and *S. cavolei* (B) during fermentative growth on 40 mM pyruvate measured in the fermentation apparatus. The graph includes the third biological independent replicate and corresponds to Figure 3 of the main manuscript.

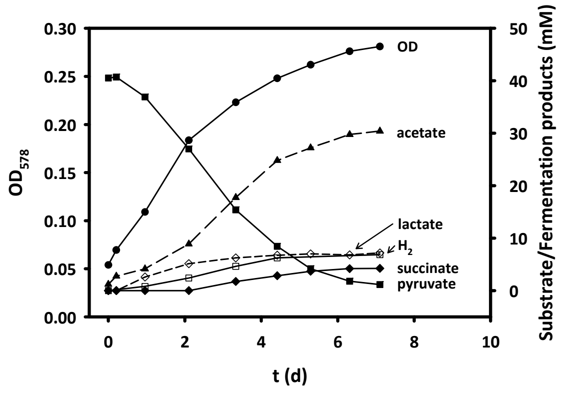

Supplementary Figure 9: Growth, substrate concentration and fermentation products of *S. deleyianum* during fermentative growth on pyruvate. Organic acids were measured via HPLC and H_2 was determined volumetrically (for details see materials and methods).

Supplementary Table 1: Oxidation/Reduction (O/R) balance and carbon recovery of pyruvate fermentation in *S. multivorans* and *S. cavolei*.

		O/R balance *S. multivorans*			O/R balance *S. cavolei*			Carbon recovery[b]		
**Substrate or Product**	O/R value[a]	mol/40 mol of substrate	O/R value mol reduced	O/R value mol oxidized	mol/40 mol of substrate	O/R value mol reduced	O/R value mol oxidized	Sum formula	*S. multivorans* carbon (mol)	*S. cavolei* carbon (mol)
**Substrate**										
**pyruvate**	0	40	-	-	40	-	-	$C_3H_4O_3$	120	120
**Products**										
**acetate**	0	27	-	-	38	-		$C_2H_4O_2$	54	76
**lactate**	-1	10	-10	-	-	-	-	$C_3H_6O_2$	30	-
**succinate**	-2	3	-6	-	-	-	-	$C_4H_6O_2$	12	-
**H_2**	-1	10	-10	-	36	-36	-	H_2	0	0
**CO_2**	+1	27	-	+27	38	-	+38	CO_2	27	38
**Total**			-26	+27		-36	+38		123	114
**O/R balance**		$\frac{\Sigma\,oxidized}{\Sigma\,reduced} = \frac{27}{26} = \underline{1.03}$			$\frac{\Sigma\,oxidized}{\Sigma\,reduced} = \frac{38}{36} = \underline{1.05}$					
**Carbon recovery**								$\frac{\Sigma\,C\,products}{\Sigma\,C\,substrate} \cdot 100$	<u>102.5%</u>	<u>95%</u>

[a]each excess of 2[H] is counted as -1; each deficiency of 2[H] is counted as +1; the O/R values for substrate oxidation and product reduction are in both organisms the same.
[b]total carbon was calculated by multiplying the number of carbons by the number of moles of each compound.

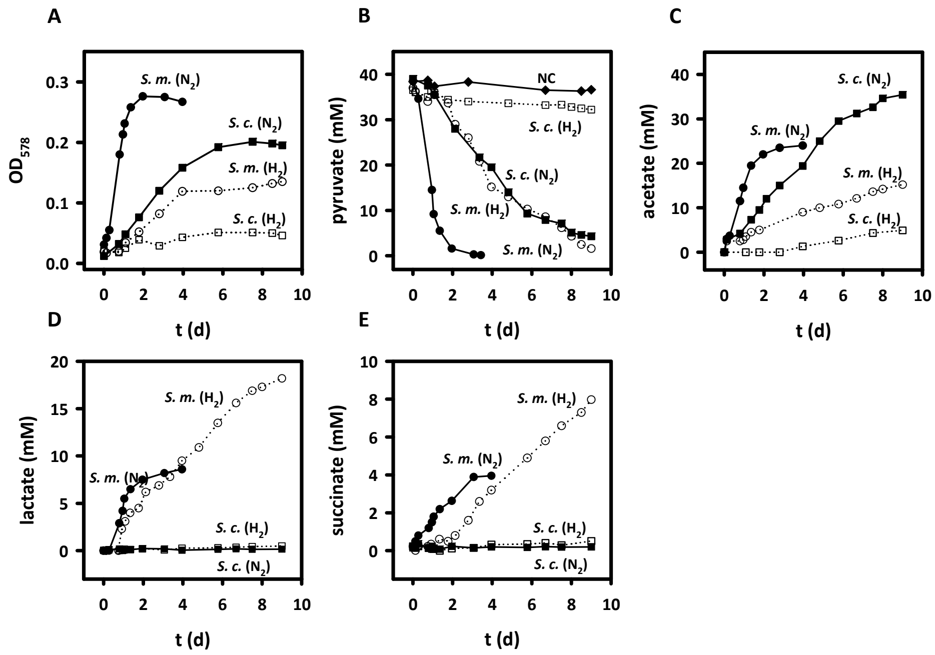

Supplementary Figure 10: Growth and formation of fermentation products during cultivation under 100% nitrogen (N_2) and 100% H_2 atmosphere with pyruvate as sole energy source. Growth curve (A), pyruvate consumption (B) and acetate (C), lactate (D) and succinate (E) production are shown. Organic acids were measured via HPLC. The graph shows the second biological independent replicate and corresponds to Figure 4 of the main manuscript. S.m. - *S. multivorans*, S.c. - *S. cavolei*, N_2 - nitrogen, H_2 - hydrogen, NC - negative control (cell-free medium).

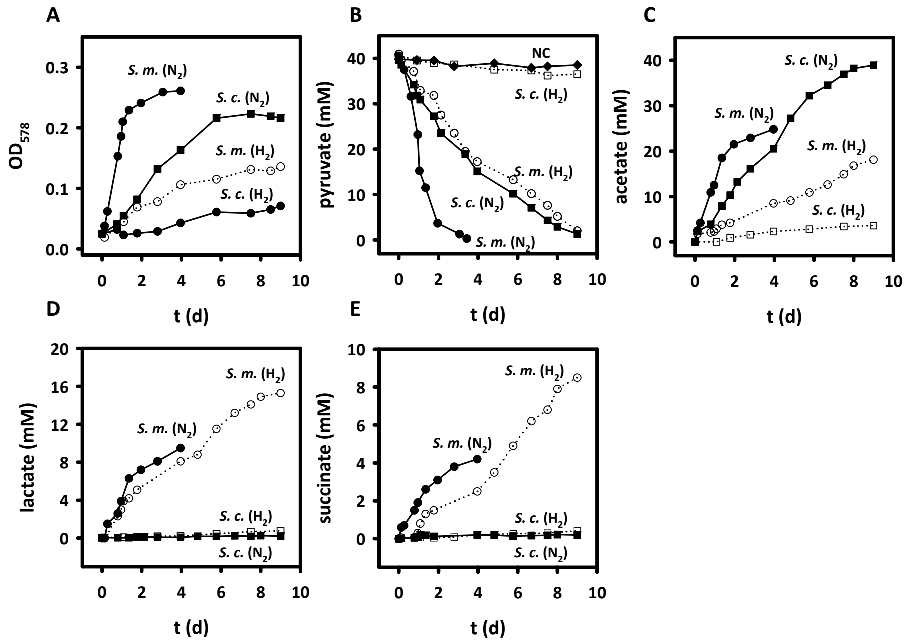

Supplementary Figure 11: Growth and formation of fermentation products during cultivation under 100% nitrogen (N_2) and 100% H_2 atmosphere with pyruvate as sole energy source. Growth curve (A), pyruvate consumption (B) and acetate (C), lactate (D) and succinate (E) production are shown. Organic acids were measured via HPLC. The graph includes the third biological independent replicate and corresponds to Figure 4 of the main manuscript. S.m. - *S. multivorans*, S.c. - *S. cavolei*, N_2 - nitrogen, H_2 - hydrogen, NC - negative control (cell-free medium).

Supplementary Table 2: Hydrogen production and oxidizing activities of cell suspensions and subcellular fractions of *C. pasteurianum* W5 grown with glucose. Data are derived from three independent biological replicates, standard deviation is given (±). MV - methyl viologen, BV - benzyl viologen, n.d. - not determined.

Cellular fraction	Hydrogenase activity (nkat/mg)		
	MV → H_2	H_2 → BV	H_2 → MV
Cell suspensions	< 0.01	3.5 ± 0.7	0.8 ± 0.1
Membrane fraction	40.3 ± 3.9	38.4 ± 0.1	n.d.
Soluble fraction	241.3 ± 25.7	314.3 ± 12.6	n.d.

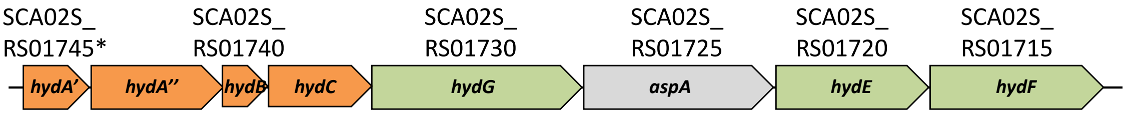

Supplementary Figure 12: [FeFe] hydrogenase gene cluster of *S. cavolei*. Point mutation in *hydA* leads to disruption of the gene into *hydA'* and *hydA''*. Locus tags of genes are given above the genes. * - hydA is annotated as pseudogene. Orange – [FeFe] hydrogenase structural genes, green - hydrogenase maturation genes, grey - not related to hydrogenase.

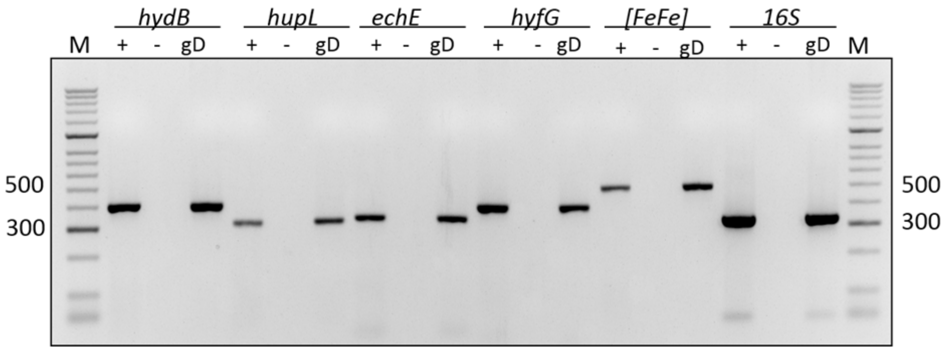

Supplementary Figure 13: Transcriptional analysis of catalytic subunits of [NiFe] hydrogenases in *S. cavolei*. *hydB* - membrane-bound hydrogenase (MBH), *hupL* - cytoplasmic uptake hydrogenase, *echE* - Ech-like hydrogenase, *hyfG* - Hyf hydrogenase, [FeFe] - [FeFe] hydrogenase *hydA*, 16S - 16S rRNA, + with reverse transcriptase, - without reverse transcriptase (negative control), gD - genomic DNA, M - DNA marker.

Supplementary Table 3: Protein abundance ratios in *S. multivorans* and *S. cavolei* cultivated with pyruvate compared to pyruvate/fumarate. Sorted after putative functional category, locus tags (hydrogenases) and abundance (other proteins). The complete dataset of proteins is given in Supplementary Dataset 1. n.d. - not quantified in the respective proteome. Magenta: significantly higher abundance, green: significantly lower abundance in the proteomes of pyruvate-cultivated cells when compared to pyruvate/fumarate-cultivated cells.

S. multivorans	***S. cavolei***	**Description**	***S. multivorans***		***S. cavolei***	
**Locus tag**	**Locus tag**		**Fold-change**	**p-value**	**Fold-change**	**p-value**
**Hydrogenase and hydrogenase accessory proteins**						
**Ech gene cluster**						
SMUL_1306	SCA02S_RS12020	soluble, cytoplasmic Ech-like hydrogenase electron transfer subunit CooF-like	only Pyr/Fum		n.d.	
SMUL_1307	SCA02S_RS12030	soluble, cytoplasmic Ech-like hydrogenase large subunit CooH-like	only Pyr/-		+ 1.0	0.807
SMUL_1308	SCA02S_RS12025	soluble, cytoplasmic Ech-like hydrogenase CooU-like	n.d.		+ 1.5	0.631
SMUL_1309	SCA02S_RS12035	soluble, cytoplasmic Ech-like hydrogenase FeS protein CooX-like	n.d.		only in Pyr/-	
SMUL_1310	SCA02S_RS12015	soluble, cytoplasmic Ech-like hydrogenase electron transfer subunit CooL-like	n.d.		only in Pyr/-	
**MBH and accessory gene cluster**						
SMUL_1423	SCA02S_RS01360	membrane-bound NiFe hydrogenase small chain precursor	- 2.0	0.400	- 3.4	<0.001
SMUL_1424	SCA02S_RS01355	membrane-bound NiFe hydrogenase large subunit	- 1.7	0.070	- 2.2	<0.001
SMUL_1425	SCA02S_RS01350	membrane-bound NiFe hydrogenase, cytochrome b subunit	+ 2.8	0.011	- 2.8	0.007
SMUL_1426	SCA02S_RS01345	NiFe hydrogenase maturation protease HydD	- 4.7	0.070	- 1.3	0.129
SMUL_1427	SCA02S_RS01340	NiFe hydrogenase accessory protein HydE	+ 1.4	0.347	- 1.5	0.081
SMUL_1428	SCA02S_RS01335	NiFe hydrogenase metallocenter assembly protein HypF	n.d.		n.d.	
SMUL_1433	SCA02S_RS00525	maturation protein HypB	- 1.0	0.718	- 1.0	0.186
SMUL_1434	SCA02S_RS00520	maturation protein HypC	only Pyr/Fum		n.d.	
SMUL_1435	SCA02S_RS00515	maturation protein HypD	- 1.6	0.027	- 1.2	0.379
SMUL_1436	SCA02S_RS00510	maturation protein HypE	- 1.6	0.123	+ 1.1	0.836
SMUL_1437	SCA02S_RS00505	maturation protein HypA	n.d.		n.d.	
**Hyf and accesory gene cluster**						
SMUL_2383	SCA02S_RS01920	hydrogenase-4 component A, iron-sulfur cluster containing subunit	+ 9.5	<0.001	+ 4.9	0.002
SMUL_2384	SCA02S_RS01925	hydrogenase-4 component B, membrane subunit	+ 27.4	<0.001	+ 4.2	0.028
SMUL_2385	SCA02S_RS01930	hydrogenase-4 component C, membrane subunit	+ 10.4	<0.001	+ 2.7	0.079
SMUL_2386	SCA02S_RS01935	hydrogenase-4 component E, membrane-subunit	only Pyr/-		n.d.	
SMUL_2387	SCA02S_RS01940	hydrogenase-4 component F, membrane-subunit	only Pyr/-		n.d.	
SMUL_2388	SCA02S_RS01945	hydrogenase-4 component G, large subunit	+ 5.5	<0.001	+ 4.3	<0.001
SMUL_2389	SCA02S_RS01950	hydrogenase-4 component H	+ 13.5	<0.001	+ 4.9	0.035
SMUL_2390	SCA02S_RS01955	hydrogenase-4 component I, small subunit	+ 7.9	<0.001	+ 4.6	<0.001
SMUL_2391	SCA02S_RS01960	hydrogenase-4 component J, accessory protein	+ 5.9	0.006	+ 1.8	0.023
SMUL_2392	SCA02S_RS01965	hydrogenase-4 component K, maturation protease	+ 4.0	0.020	+ 7.2	<0.001
**Other proteins possibly involved in the energy metabolism**						
		**Sorted by *S. multivorans***				
SMUL_2101	SCA02S_RS10975	aldehyde oxidoreductase	+ 141.6	<0.001	+ 6.6	<0.001
SMUL_2394	SCA02S_RS05380	aldehyde ferredoxin oxidoreductase	+ 8.2	<0.001	- 3.2	0.062
SMUL_0303	SCA02S_RS12260	Ferredoxin	+ 6.5	0.008	+ 2.4	0.013
SMUL_1602	/	phosphoenolpyruvate synthase	+ 6.3	0.021	/	

SMUL_2522	SCA02S_RS04100	ubiquinol--cytochrome c reductase, cytochrome B subunit	+ 4.6	0.042	+ 1.5	0.043
SMUL_2901	SCA02S_RS08980	formate dehydrogenase, catalytic subunit alpha FdhA	+ 4.5	0.037	n.d.	<0.001
SMUL_3158	SCA02S_RS00475	NADP-dependent malic enzyme	+ 3.7	<0.001	/	/
SMUL_2630	SCA02S_RS04525	pyruvate-ferredoxin/flavodoxin oxidoreductase	+ 2.0	0.007	+ 3.8	<0.001
		**Sorted by *S. cavolei***				
SMUL_1318	SCA02S_RS11745	(Fe-S)-binding protein	+ 1.7	0.027	+ 8.9	<0.001
SMUL_1317	SCA02S_RS11750	lactate utilization protein	+ 1.2	0.475	+ 8.4	<0.001
SMUL_3281	SCA02S_RS07425	molybdopterin oxidoreductase	- 3.3	0.179	+ 3.9	<0.001
SMUL_0787	SCA02S_RS02530	FAD-binding oxidoreductase	- 1.9	0.002	+ 3.2	<0.001
SMUL_1484	SCA02S_RS00240	acetate kinase	+ 1.7	0.005	+ 3.2	<0.001
SMUL_1483	SCA02S_RS00245	phosphate acetyltransferase	+ 2.0	0.003	+ 3.1	<0.001
SMUL_1698	SCA02S_RS08870	LLM class flavin-dependent oxidoreductase	1.0	0.801	+ 2.6	0.001
/	SCA02S_RS01635	NAD(P)-dependent alcohol dehydrogenase	/		+ 2.2	0.006
		**Sorted by *S. multivorans***				
SMUL_1640	SCA02S_RS00755	FAD-dependent oxidoreductase	- 12,1	<0.001	- 1.2	0.062
SMUL_1703	/	pyruvate dehydrogenase [ubiquinone]	- 6.8	0.016	/	
SMUL_1531	/	tetrachloroethene reductive dehalogenase catalytic subunit PceA	- 6.4	0.022	/	
SMUL_1365	SCA02S_RS09935	putative NAD(P)H nitroreductase	- 6.3	<0.001	+ 1.1	0.993
SMUL_2873	SCA02S_RS05345	formate dehydrogenase, subunit alpha FdhA	- 5.1	<0.001	- 12.9	<0.001
SMUL_2872	SCA02S_RS05340	formate dehydrogenase, iron-sulfur subunit FdhB	- 4.3	<0.001	- 15.7	<0.001
SMUL_0200	SCA02S_RS07925	epsilonproteobacterial nuoF-like protein	- 4.3	<0.001	+ 1.5	0.143
SMUL_1459	SCA02S_RS08715	fumarate hydratase class II	- 4.2	0.001	+ 2.5	0.072
SMUL_3037	SCA02S_RS10055	acetaldehyde dehydrogenase	- 3.9	0.002	+ 1.3	0.976
SMUL_0196	SCA02S_RS06700	NADH-ubiquinone oxidoreductase chain B	- 2.9	<0.001	- 1.2	0.047
SMUL_0203	SCA02S_RS06665	NADH-ubiquinone oxidoreductase chain I	- 2.8	0.011	- 1.6	0.017
SMUL_0197	SCA02S_RS06695	NADH-ubiquinone oxidoreductase chain C	- 2.5	<0.001	- 1.4	0.053
SMUL_0195	SCA02S_RS06705	NADH-ubiquinone oxidoreductase chain A	- 2.5	<0.001	n.d.	
SMUL_0198	SCA02S_RS06690	NADH-ubiquinone oxidoreductase chain D	- 2.2	0.002	- 1.5	0.006
		**Sorted by *S. cavolei***				
SMUL_0552	SCA02S_RS07735	fumarate reductase	- 1.2	0.213	- 6.9	<0.001
SMUL_1680	SCA02S_RS00615	fumarate hydratase	- 1.8	0.015	- 6.0	<0.001
SMUL_1679	SCA02S_RS00620	fumarate hydratase	- 2.2	0.013	- 5.7	<0.001
SMUL_0551	SCA02S_RS07740	fumarate reductase flavoprotein subunit	- 1.1	0.285	- 5.3	<0.001
SMUL_0199	SCA02S_RS06685	NADH-ubiquinone oxidoreductase subunit E	n.d.		- 2.9	0.002
**Other proteins**						
		**Sorted by *S. multivorans***				
SMUL_1016	SCA02S_RS06075	heavy metal resistance P-type ATPase	+ 39.8	<0.001	- 2.8	0.023
SMUL_1017	SCA02S_RS09930	heavy metal efflux system accessory protein	+ 19.2	<0.001	only Pyr/-	
SMUL_2587	SCA02S_RS04335	phosphate ABC transporter, periplasmic phosphate-binding protein	+ 17.5	0.002	n.d.	
SMUL_0955	SCA02S_RS01815	putative two-component sensor histidine kinase	+ 13.5	<0.001	only Pyr/-	
SMUL_0188	SCA02S_RS06740	manganese/zinc/iron chelate uptake transporter (MZT) family	+ 8.9	<0.001	n.d.	
SMUL_1512	SCA02S_RS00550	PHP/AAA ATPase domain-containing protein	+ 7.3	<0.001	+ 1.5	0.269
SMUL_3232	SCA02S_RS09470	NosL family protein	+ 5.9	0.042	+ 6.1	<0.001
SMUL_0547	SCA02S_RS07760	heat shock protein Hsp20	+ 5.5	0.004	- 1.2	0.232

		**Sorted by *S. cavolei***				
SMUL_2124	SCA02S_RS05290	cytochrome c	only Pyr/-		+ 92.9	<0.001
SMUL_0325	SCA02S_RS12370	homoserine kinase	+ 1.5	0.277	+ 10.6	0.039
SMUL_0337	SCA02S_RS12425	cytochrome c family protein	+ 0.7	0.132	+ 5.2	<0.001
SMUL_2091	SCA02S_RS11025	HlyC/CorC family transporter	- 1.2	0.251	+ 4.6	0.002
SMUL_1239	SCA02S_RS12085	Hydrolase	n.d.		+ 4.3	0.008
SMUL_0313	SCA02S_RS12310	sensor histidine kinase	+ 1.7	0.310	+ 4.2	<0.001
SMUL_2235	SCA02S_RS12725	type II secretion system protein	n.d.		+ 3.9	0.021
SMUL_0529	SCA02S_RS07855	superoxide dismutase	- 1.4	0.121	+ 3.2	<0.001
		**Sorted by *S. multivorans***				
SMUL_0622	SCA02S_RS03235	TRAP-type C4-dicarboxylate transport system, periplasmic component	- 12.3	<0.001	- 3.4	<0.001
SMUL_1639	SCA02S_RS00760	cytochrome c	- 10.2	<0.001	- 2.3	0.654
SMUL_0306	SCA02S_RS12275	50S ribosomal protein L32	- 6.6	<0.001	- 2.2	0.005
SMUL_0892	SCA02S_RS02045	nitrite reductase cytochrome c biogenesis protein NrfJ	- 6.1	0.017	n.d.	
SMUL_0802	SCA02S_RS02460	RND family protein	- 4.6	0.010	only Pyr/Fum	
SMUL_0804	SCA02S_RS02450	RND family protein	- 4.2	<0.001	only Pyr/Fum	
SMUL_0224	SCA02S_RS06575	LSU ribosomal protein L31p	- 4.1	0.002	- 2.3	0.006
SMUL_2691	SCA02S_RS04835	SSU ribosomal protein S14p (S29e), Zinc-independent	- 3.8	0.012	- 1.9	<0.001
SMUL_0477	SCA02S_RS08145	LSU ribosomal protein L33p	- 3.8	0.001	- 1.6	0.003
SMUL_3236	SCA02S_RS09455	RNP-1 like RNA-binding protein	- 3.3	0.001	- 1.5	<0.001
SMUL_0987	SCA02S_RS14055	heat shock protein 60 family co-chaperone GroES	- 3.6	0.003	- 2.0	0.002
SMUL_1429	SCA02S_RS01330	nickel responsive regulator NikR	- 2.9	<0.001	+ 1.1	0.679
SMUL_2699	SCA02S_RS04875	LSU ribosomal protein L22p (L17e)	- 2.9	<0.001	- 2.0	<0.001
SMUL_2696	SCA02S_RS04860	LSU ribosomal protein L29p (L35e)	- 2.8	0.004	- 2.4	0.004
SMUL_2290	SCA02S_RS12980	SSU ribosomal protein S16p	- 2.7	0.006	- 1.4	0.085
		**Sorted by *S. cavolei***				
SMUL_1277	SCA02S_RS12160	molybdopterin guanine dinucleotide-containing S/N-oxide reductase	n.d.		- 35.0	<0.001
SMUL_2591	SCA02S_RS00610	citrate:succinate antiporter	n.d.		- 32.6	<0.001
SMUL_0963	SCA02S_RS01780	phosphatidylserine decarboxylase	- 1.1	0.843	- 3.6	0.002
SMUL_2857	SCA02S_RS08830	sulfurtransferase-like selenium metabolism protein YedF	- 3.3	0.001	- 3.6	<0.001
SMUL_0851	SCA02S_RS02250	DNA-directed RNA polymerase subunit omega	- 4.0	0.004	- 3.1	0.034
SMUL_0141	SCA02S_RS06970	30S ribosomal protein S21	- 1.9	0.015	- 2.6	<0.001
SMUL_1944	SCA02S_RS11615	30S ribosomal protein S15	- 1.9	0.002	- 2.3	<0.001
SMUL_0482	SCA02S_RS08125	50S ribosomal protein L1	- 1.5	0.020	- 2.1	<0.001

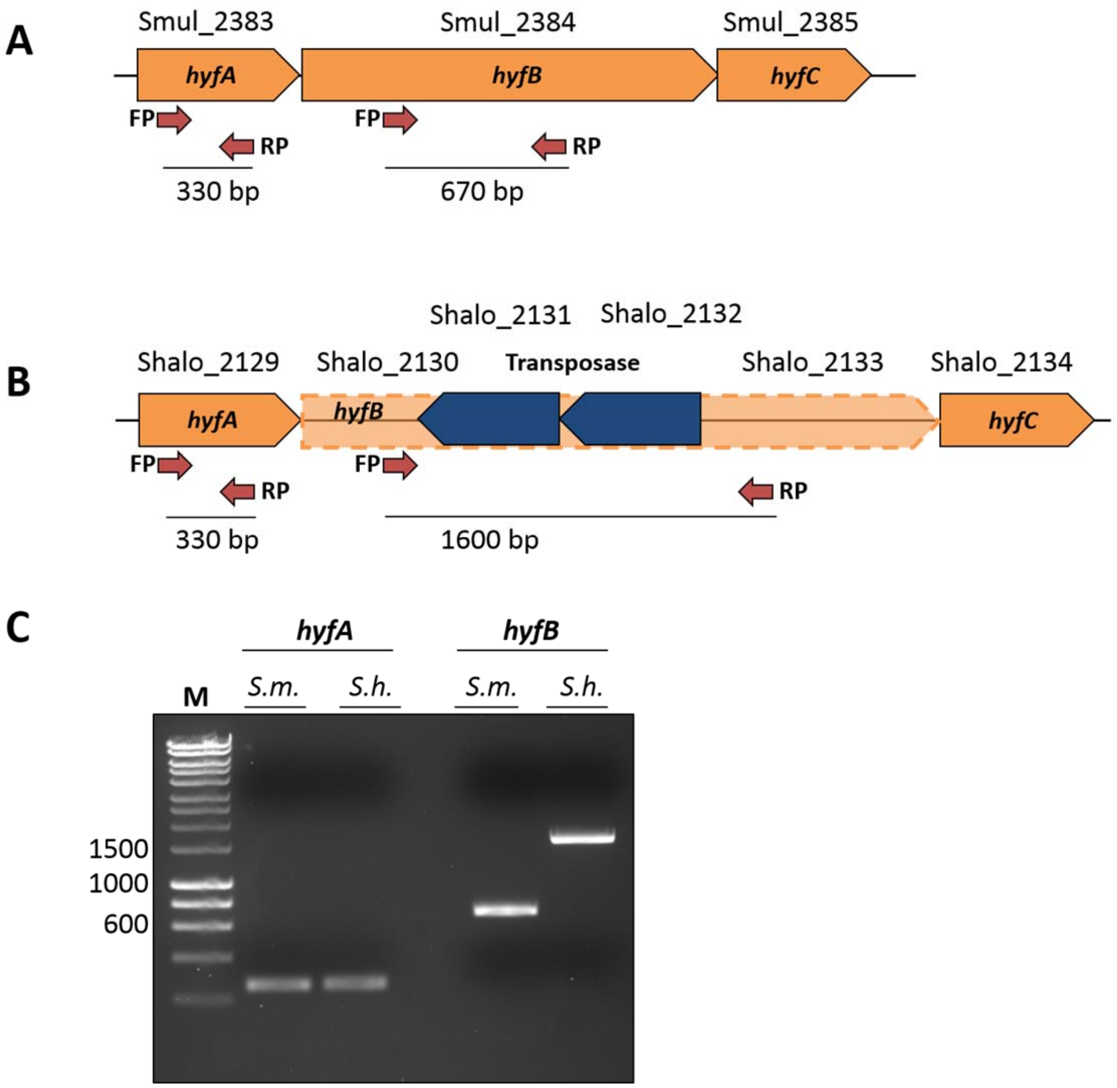

Supplementary Figure 14: Hyf-like hydrogenase gene cluster of *S. multivorans* (A) and *S. halorespirans* (B) and confirmation of transposon integration in *hyfB* of *S. halorespirans* (C). (A, B) Locus tags are given above the genes and primer binding sites are indicated by red arrows. Transposase in *hyfB* of *S. halorespirans* coloured dark blue. Structural genes are coloured orange. (C) PCR with primers binding in *hyfA* and *hyfB* of *S. multivorans* and *S. halorespirans*. M - marker lane, *S.m.* - *S. multivorans*, *S.h.* - *S. halorespirans*, NiFe - catalytic subunit. Primer used are listed in Supplementary Table 4.

Supplementary Table 4: Oligonucleotides used for transcriptional analysis of hydrogenase catalytic subunits in *S. cavolei* (Supplementary Figure 6) and PCR of *hyfA* and *hyfB* of *S. multivorans* and *S. halorespirans* (Supplementary Figure 8).

Primer	Sequence (5' – 3')	Amplicon size (bp)
S. cavolei		
*hydB*_fw	GTATAAGTTAACGCCTGAGCAA	402
*hydB*_rev	CCACTCACGAGATTAATGACG	
*hupL*_fw	GGATTTGATTCCGCCGTA	324
*hupL*_rev	TCCCTCTTTGGCTACCATC	
*echE*_fw	ACCGACGAGATCAAACGTATC	339
*echE*_rev	GAGTTTGAGGGCATCTTCATAC	
*hyfG*_fw	CGCTTGGTCTTAAGCGAC	398
*hyfG*_rev	GGAATTTCCAAGTTGATCGC	
*[FeFe]*_fw	CTAACAAATGCGTCGCGT	504
*[FeFe]*_rev	TCCATACAAACTCCGCCG	
*16S*_fw	GAGACACGGTCCAGACTCCTAC	334
*16S*_rev	CACCAATTCCATCTACCTCTCCC	
S. multivorans		
*hyfA*_fw	CCCAACCAGTGTCGTCAA	285
*hyfA*_rev	CGTGTATAGCGTAAACTACC	
*hyfB*_fw	ATGACATTAATATCCGCACT	672
*hyfB*_rev	CATATCGCGTGTTTTGGTTG	
S. halorespirans		
*hyfA*_fw	CCCAACCAGTGTCGTCAA	285
*hyfA*_rev	CGTGTATAGCGTAAACTACC	
*hyfB*_fw	ATGACATTAATATCCGCACT	1585
*hyfB*_rev	CATATCGCGTGTTTTGGTTG	

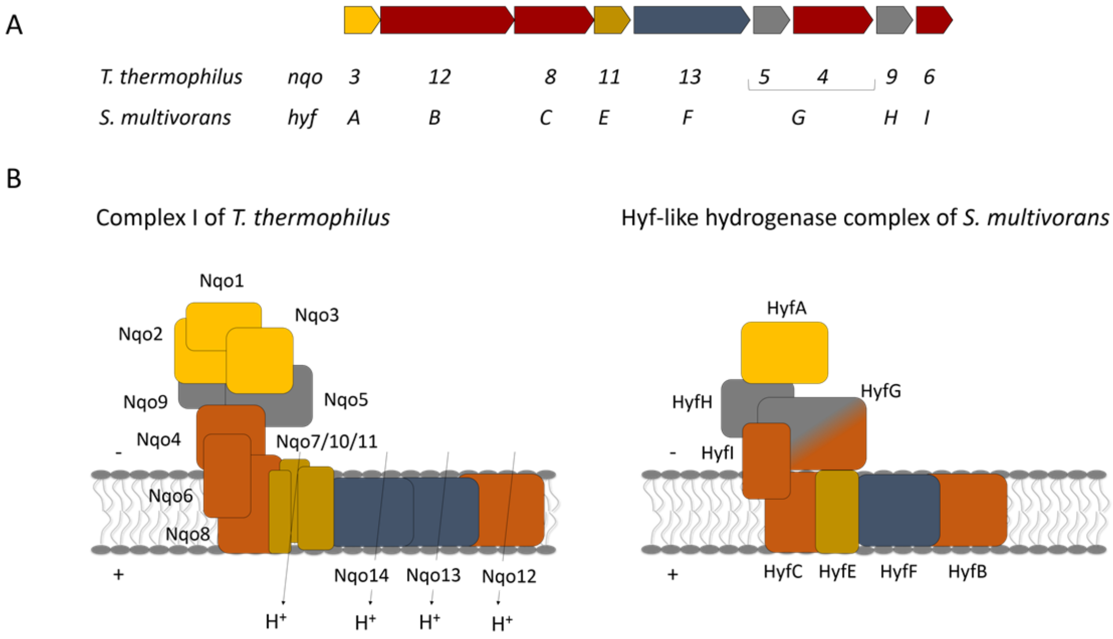

Supplementary Figure 15: Comparison of Hyf-like hydrogenase of *S. multivorans* to complex I of *Thermus thermophilus*. (A) Schematic representation of genetic organization. Subunits of complex I (Nqo) are directly compared to subunits of Hyf-like hydrogenase (hyf). Nqo5 and Nqo4 are fused to HyfG. (B) Hypothetical scheme of the structural organization of both complexes. Arrows indicate putative proton-translocation channels in *T. thermophiles* complex I. Color code resembles corresponding homologues.

SUPPLEMENTARY NOTE 1

Hyf-like hydrogenase of *Sulfurospirillum* spp. as a proton pump

The relationship of the subunit composition and amino acid sequences of group 4 hydrogenases to the respiratory complex I and the possible involvement of these membrane-bound hydrogenases in energy conservation via the generation of a proton motive force has been discussed previously[1]. In *S. multivorans* and other *Sulfurospirillum* spp., the eight subunits of Hyf (HyfABCEFGHI) are homologs to the subunits of complex I (Supplementary Figure 15, Supplementary Table 5). The most likely candidate for potential H^+-transport HyfF, four putative key amino acids for proton transfer, Glu148, Lys237, Lys268, and Glu424 are present (Supplementary Figures 16-18). The high conservation grade of key amino acid residues possibly involved in proton transfer especially in HyfB renders the involvement of *Sulfurospirillum* spp. Hyf in energy conservation via a chemosmotic generation of ATP a possible scenario.

Supplementary Table 5: Comparison of complex I subunits to homologs in bacteria.

Gene order	Complex I (Eukaryotes)	*E. coli* complex I	*T. thermophilus* complex I	*S. multivorans* (Hyf)	*E. coli* (Hyf/Hyc)
**1**	ND3	NuoA	Nqo7	-	-
**2**	IP21K	NuoB	Nqo6	HyfI	HyfI/HycG
**3**	IP30K	NuoC	Nqo5[1]	HyfG	HyfG/HycE
**4**	IP49K	NuoD	Nqo4[1]		
**5**	IP24K	NuoE	Nqo2	-	-
**6**	IP51K	NuoF	Nqo1	-	-
**7**	IP75K	NuoG	Nqo3	HyfA	HyfA/HycB
**8**	ND1	NuoH	Nqo8	HyfC	HyfC/HycD
**9**	IP23K	NuoI	Nqo9	HyfH	HyfH/HycF
**10**	ND6	NuoJ	Nqo10	-	-
**11**	ND4L	NuoK	Nqo11	HyfE	HyfE/-
**12**	ND5	NuoL	Nqo12	HyfB	HyfB/HyfD/HycC
**13**	ND4	NuoM	Nqo13	HyfF	HyfF/HycC
**14**	ND2	NuoN	Nqo14	-	-

[1]Nqo5 and Nqo4-similar proteins are fused to HyfG.

```
                10        20        30        40        50        60        70
            ....|....|....|....|....|....|....|....|....|....|....|....|....|....|
S. mul      ------MQTIYTLFLLTSLLSLALYKKPLLAQKIGFG---LSSLISLYAAIFFFSHLG-ETMTWQLPGNF
E. coli4    MDALQLLTWSLILYLFASLASLFLLGLDRLAIKLSGITSLVGGVIG-IISGITQLHAGVTLVARFAPPFE
E. coli3    MSAISLINSGVAWFVAAAVLAFLFSFQKALSGWIAGIGGAVGSLYT-AAAGFTVLTGAVGVSG----ALS
T. ther     ------MALLGTILLPLLGFALLGLFGKRMREPLPGVLASGLVLASFLLGAGLLLSGGARFQAEWLPGIP
B. tau      MNMFSSLSLVTLLLLTMPIMMMSFNTYKPSNYPLYVKTAISYAFITSMIPTMMFIHSGQELIISNWHWLT
O. aries    MNLFSSLTLVTLILLTMPIAAINFNTHKFTNYPLYVKTTISCAFITSMIPTMMFIHTGQEMIISNWHWLT

                80        90       100       110       120       130       140
            ....|....|....|....|....|....|....|....|....|....|....|....|....|....|
S. mul      ISSPLFRLDSIEMFFSFLVSLIAFAVSLFSFDYAKFYEKKAN-LAVFASLFNAFILSMLLVIASDNVFSF
E. coli4    FADLTLRMDSLSAFMVLVISLLVVVCSLYSLTYMREYEGKGAAAMGFFMN--IFIASMVALLVMDNAFWF
E. coli3    LVSYDVQISPLNAIWLITLGLCGLFVSLYNIDWHRHAQVK---CNGLQIN--MLMAAAVCAVIASNLGMF
T. ther     FS---LLLDNLSGFMLLIVTGVGFLIHVYAIG----YMGGDPGYSRFFAYFNLFIAMMLTLVLADSYPVM
B. tau      IQTLKLSLSFKMDYFSMMFIPVALFVTWSIMEFSMWYMYSDPNINKFFKYLLLFLITMLILVTANNLFQL
O. aries    IQTLKLSLSFKMDFFSMMFVPVALFVTWSIMEFSMWYMHSDPNINQFFKYLLLFLITMLILVTANNLFQL

               150       160       170       180       190       200       210
            ....|....|....|....|....|....|....|....|....|....|....|....|....|....|
S. mul      MLLWEMMTLISALLILINDGEGAGKKVMIYLGIA-QIGASCLMVALLIMASFAGSFEFSKFADLNIGFGM
E. coli4    IVLFEMMSLSSWFLVIARQDKTSINAGMLYFFIA-HAGSVLIMIAFLLMGRESGSLDFASFRTLSLSPGL
E. coli3    VVMAEIMALCAVFLTSNSKE------GKLWFALG-RLGTLLLAIACWLLWQRYGTLDLRLLDMRMQQLPL
T. ther     FIGWEGVGLASFLLIGFWYKNPQYADSARKAFIVNRIGDLGFMLGMAILWALYGTLSISELKEAMEGPLK
B. tau      FIGWEGVGIMSFLLIGWWYGRADANTAALQAILYNRIGDIGFILAMAWFLTNLNTWDLQQIFMLNPSD--
O. aries    FIGWEGVGIMSFLLIGWWYGRTDANTAALQAILYNRIGDIGFILAMAWFLINLNTWDLQQIFMLNPND--

               220       230       240       250       260       270       280
            ....|....|....|....|....|....|....|....|....|....|....|....|....|....|
S. mul      SITLFTLL----LVGLGSKAGMFPFHVWLPLAYCQSPSNASALMSGVMIKVALFAFIKFSLLLPQFA---
E. coli4    ASAVFLLA----FFGFGAKAGMMPLHSWLPRAHPAAPSHASALMSGVMVKIGIFGILKVAMDLLAQTGLP
E. coli3    GSDIWLLG----VIGFGLLAGIIPLHGWVPQAHANASTPAAALFSTVVMKIGLLGILTLS--LLGGN-AP
T. ther     NPDLLALAGLLLFLGAVGKSAQIPLMVWLPDAMAGPTPVSALIHAATMVTAGVYLIARSSFLYSVLP---
B. tau      SNMPLIGL----ALAATGKSAQFGLHPWLPSAMEGPTPVSALLHSSTMVVAGIFLLIRFYPLTENNK---
O. aries    SNLPLMGL----ILAATGKSAQFGLHPWLPSAMEGPTPVSALLHSSTMVVAGIFLLIRFYPLTENNK---

               290       300       310       320       330       340       350
            ....|....|....|....|....|....|....|....|....|....|....|....|....|....|
S. mul      -QFGYILLFMGALSCIFGIIYALASNDYKASIAYSSCENVGIIFLGLGGAFYGLGINSPMIALMGFIAAF
E. coli4    LWWGILVMAIGAISALLGVLYALAEQDIKRLLAWSTVENVGIILLAVGVAMVGLSLHDPLLTVVGLLGAL
E. coli3    LWWGIALLVLGMITAFVGGLYALVEHNIQRLLAYHTLENIGIILLGLGAGVTGIALEQPALIALGLVGGL
T. ther     -DVSYAIAVVGLLTAAYGALSAFGQTDIKKIVAYSTISQLGYMFLAAGVGAY--------------WVAL
B. tau      -YIQSITLCLGAITTLFTAMCALTQNDIKKIIAFSTSSQLGLMMVTIGINQP--------------YLAF
O. aries    -FGQSIMLCLGAMTTLFTAMCALTQNDIKKIIAFSTSSQLGLMMVTIGINQP--------------HLAF

               360       370       380       390       400       410       420
            ....|....|....|....|....|....|....|....|....|....|....|....|....|....|
S. mul      FHILNHAVFKSLLFMLSGNVFTATK-TRDMDALGGLHKKMPITSIIFFVAAISICALPPLNGFASEWVIY
E. coli4    FHLLNHALFKGLLFLGAGAIISRLH-THDMEKMGALAKRMPWTAAACLIGCLAISAIPPLNGFISEWYTW
E. coli3    YHLLNHSLFKSVLFLGAGSVWFRTG-HRDIEKLGGIGKKMPVISIAMLVGLMAMAALPPLNGFAGEWVIY
T. ther     FHVFTHAFFKALLFLASGSVIHALGGEQDVRKMGGLWKHLPQTRWHALIGALALGGLPLLSGFWSKDAIL
B. tau      LHICTHAFFKAMLFMCSGSIIHSLNDEQDIRKMGGLFKAMPFTTTALIVGSLALTGMPFLTGFYSKDLII
O. aries    LHICTHAFFKAMLFMCSGSIIHSLNDEQDIRKMGGLFKAMPFTTTALIIGSLALTGMPFLTGFYSKDLII

               430       440       450       460       470       480       490
            ....|....|....|....|....|....|....|....|....|....|....|....|....|....|
S. mul      KTMVMGGIDEGVASRFFFTLAIIALSITGAMAIMAFSKMYGSVFLGIARNTKCVEEAKEVSFIRLLPLGL
E. coli4    QSLFSLSRVEAVALQLAGPIAMVMLAVTGGLAVMCFVKMYGITFCGAPR-STHAEEAQEVPNTMIVAMLL
E. coli3    QSFFKLSNSGAFVARLLGPLLAVGLAITGALAVMCMAKVYGVTFLGAPR-TKEAENATCAPLLMSVSVVA
T. ther     AATLT--------YPFGGVGFYVGALLVAVLTAMYAMRWFVLVFLGEER---GHHHPHEAPPVMLWPNHL
B. tau      EAAN---------TSYTNAWALLMTLIATSFTAIYSTRIIFFALLGQPR-FPTLVNINENNPLLINSIKR
O. aries    ESAN---------TSYTNAWALLMTLVATSFTAIYSTRIIFFALLGQPR-FPTLININENNPFLINSIKR

               500       510       520       530       540       550       560
            ....|....|....|....|....|....|....|....|....|....|....|....|....|....|
S. mul      LASLCVGIG-IFMSDVVG-----------MLSKIVLTLIPQTSQSVFGLISMPIIIMIMLLCAIIPFVFL
E. coli4    LAALCVLIA-LSASWLAPKIMHIAHAFTNTPPATVASGIALVPGTFHTQVTPSLLLLLLLAMPLLPGLYW
E. coli3    LAICCVIGG-VAAPWLLP---------------MLSAAVPLPLEPANTTVSQPMITLLLIACPLLPFIIM
T. ther     LALGSVLAGYLALPHPLPN---------VLEPFLKPALAEVEAHHLSLGAEWGLIALSAAVALLGLWAGF
B. tau      LLIGSLFAG-YIISNNIP-----------------PTTIPQMTMPYYLKTTALIVTILGFILALEISNMT
O. aries    LLIGSLFAG-FIISNNIP-----------------PMTIPQMTMPHYLKMTALTVTILGFILALEISNTT
```

```
             570       580       590       600       610       620       630
          ....|....|....|....|....|....|....|....|....|....|....|....|....|....|
S. mul    YLLKAN-HKEVRVTEPWACGFLYNKNMQIGSNSFTGDIRKALSFILRHEQEIKIDGYFS-----KAVYTQ
E. coli4  LWCRSRRAAFRRTGDAWACGYGWENAMAPSGNGVMQPLRVVFSALFRLRQQLDPTLRLNKGLAHVTARAQ
E. coli3  AICKGDRLPSRSRGAAWVCGYDHEKSMVITAHGFAMPVKQAFAPVLKLRKWLNP----------------
T. ther   VFFQRKVFPAWYLAFEAASREAFYVDRAYNALIVNPLKALAEALFYGDRGLLSGYFGLG-----------
B. tau    KNLKYH-YPSNAFKFSTLLGY-FPTIMHRLAPYMNLSMSQKSASSLLDLIWLEAILP-------------
O. aries  HYLKFN-YPSNTFKFSNLLGY-YPTIMHRLTPYMNLTMSQKSASSLLDLIWLETILP-------------
```

```
             640       650       660       670       680
          ....|....|....|....|....|....|....|....|....|....|....|...
S. mul    KVHDLFWDKLYAPVIHSVMVIADKIGIFQNGRTNLYAGYILIYLCFVLIFGYYYL---
E. coli4  STEPFWDERVIRPIVSATQRLAKEIQHLQSGDFRLYCLYVVAALVVLLIAIAV-----
E. coli3  --------------VSLVPGWQCEGSALL---FRRMALVELAVLVVIIVSRGA-----
T. ther   ---------------GAARSLGQGLARLQTGYLRVYALLFVLGALLLLGVMRW-----
B. tau    ---------------KTISLAQMKASTLVTNQKGLIKLYFLSFLITILISMILFNFHE
O. aries  ---------------KTISLAQMKMSTTITSQKGLIKLYFLSFLITILISTTLLNFHE
```

Supplementary Figure 16: Amino acid sequence alignment of HyfB/Nqo12/ND5. *S. mul* - *S. multivorans* AHJ13630.1 Hyf-like hydrogenase, membrane subunit B, *E. coli*4 - *E. coli* AAB88564.1 Hyf hydrogenase, membrane subunit B, *E. coli*3 - *E. coli* CAA35548.1 - Hyc hydrogenase, membrane subunit 3, *T. ther* - *T. thermophilus* Q56227.1 NADH-quinone oxidoreductase subunit 12 (Nqo12), *B. tau* - *Bos taurus* P03920.2 NADH-ubiquinone oxidoreductase chain 5 (ND5), *O. aries* - *Ovis aries* O78756.1 NADH-ubiquinone oxidoreductase chain 5 (ND5). Amino acid color code: green - hydrophobic, red - charged, blue - polar/uncharged. Key charged residues are highlighted in yellow.

```
              10        20        30        40        50        60        70
          ....|....|....|....|....|....|....|....|....|....|....|....|....|....|
S. mul    ------MDILVLILTVPFVFGVIMFCMP----LHFKLLQSLHIVLSVAVSILLLSAVGKVVNGEELSIFH
E. coli4  --------MFALLLLTPLLFSLLCFACRKRGLSATCTVTVLHSLGITLLLILALWVVQTAADAGEIFAAG
E. coli3  MSAISLINSGVAWFVAAAVLAFLFSFQKALSGWIAGIGGAVGSLYTAAAGFTVLTGAVGVSGALSLVSYD
T. ther   --------MVVLAVLLPVVFGALLLLGLPR--ALGVLGAGLSFLLNLYLFLTHPGGVAHAFQAPLLPGAG
B. tau    --------MLKYIIPTIMLMPLTWLSKN----NMIWVNSTAHSLLISFTSLLLMNQFGDNS-----LNFS
O. aries  --------MLKYIIPTMMLMPLTWLSKN----SMIWINTTLHSLLISLTSLLLLNQFGDNS-----LNFS

              80        90       100       110       120       130       140
          ....|....|....|....|....|....|....|....|....|....|....|....|....|....|
S. mul    NYIFL--DSLGAIFLSLIAITGLLVNVYATTYMKWELEDGHIDIKEVKNYFALSFIFTWTMSLSVVCNNI
E. coli4  LWLHI--DGLGGLFLAILGVIGFLTGVYSIGYMRHEVAHGELSPVTLCDYYGFFHLFLFTMLLVVTSNNL
E. coli3  VQISP----LNAIWLITLGLCGLFVSLYNIDWHRHA--------QVKCNGLQINMLMAAAVCAVIAS-NL
T. ther   VYWAFGLDGLSALFFLTIALTVFLGALVAR---------------VEGRFLGLALLMEGLLLGLFAARDL
B. tau    LLFFS--DSLSTPLLILTMWLLPLMLMASQHHLSKE------NLTRKKLFITMLISLQLFLIMTFTAMEL
O. aries  LTFFS--DSLSTPLLILTMWLLPLMLMASQHHLSKE------NLARKKLFISMLILLQLFLIMTFTATEL

             150       160       170       180       190       200       210
          ....|....|....|....|....|....|....|....|....|....|....|....|....|....|
S. mul    AFMWAAIEATTLASVFLVAVKKDKKSTESGYKYIVLCSIGLAFALYATILLFS--AANGKIDGEAMLYTN
E. coli4  IVMWAAIEATTLSSAFLVGIYGQRSSLEAAWKYIIICTVGVAFGLFGTVLVYANVASVMPQAEMAIFWSE
E. coli3  GMFVVMAEIMALCAVFLTSN------SKEGKLWFALGRLGTLLLAIACWLLWQR------YGTLDLRLLD
T. ther   LVFYVFFEAALIPALLMLYLYGGEGRTRALYTFVLFTLVG-SLPMLAAVLGAR-----LLSGSPTFLLED
B. tau    ILFYILFEATLVPTLIIITRWGNQTERLNAGLYFLFYTLAGSLPLLVALIYIQN-----TVGSLNFLMLQ
O. aries  IFFYIMFEATLVPTLIIITRWGNQTERLNAGLYFLFYTLAGSLPLLVALIYIQN-----TMGSLNFLILQ
```

```
                220       230       240       250       260       270       280
            ....|....|....|....|....|....|....|....|....|....|....|....|....|....|
S. mul      LLANAANLDSVALKLVFIFALIGFGTKAGLAPTHTWLPDVHAEGPAPTSALLSGILLKCAMLGLIRYYAI
E. coli4    VLKQSSLLDPTLMLLAFVFVLIGFGTKTGLFPMHAWLPDAHSEAPSPVSALLSAVLLNCALLVLIRYYII
E. coli3    MRMQQLPLGSD----IWLLGVIGFGLLAGIIPLHGWVPQAHANASTPAAALFSTVVMKIGLLGILTLSLL
T. ther     LLAHP--LQEEAAFWVFLGFALAFAIKTPLFPLHAWLPPFHQENHPSGLADALGTLYKVGVFAFFRFAIP
B. tau      YWVQP-VHNSWSNVFMWLACMMAFMVKMPLYGLHLWLPKAHVEAPIAGSMVLAAVLLKLGGYGMLRITLI
O. aries    YWVQP-MPNSWSNTFMWLACMMAFMVKMPLYGLHLWLPKAHVEAPIAGSMVLAAILLKLGGYGMMRITLL

                290       300       310       320       330       340       350
            ....|....|....|....|....|....|....|....|....|....|....|....|....|....|
S. mul      VANGVGFDFVQTVMVVSGTLTLFISAFFLIRQHNVKRMFAYHSVAHMGVIAFGLGVGGA-----------
E. coli4    ICQAIGSDFPNRLLLIFGMLSVAVAAFFILVQRDIKRLLAYSSVENMGLVAVALGIGGP-----------
E. coli3    GGNAP--LWWGIALLVLGMITAFVGGLYALVEHNIQRLLAYHTLENIGIILLGLGAGVTGIALEQPALIA
T. ther     LAPEG-FAQAQGLLLFLAALSALYGAWVAFAAKDFKTLLAYAGLSHMGVAALGVFSGTP-----------
B. tau      LNPMT--DFMAYPFIMLSLWGMIMTSSICLRQTDLKSLIAYSSVSHMALVIVAILIQTP-----------
O. aries    LNPIT--DFMAYPFIMLSLWGMIMTSSICLRQTDLKSLIAYSSVSHMALVIVAILIQTP-----------

                360       370       380       390       400       410       420
            ....|....|....|....|....|....|....|....|....|....|....|....|....|....|
S. mul      IGLFAALFHCAAHSFTKALAFCSTGNIARIYGTKDMTKMGGMIRIAPLTAVLFG--IAICSLVGVPGFAI
E. coli4    LGIFAALLHTLNHSLAKTLLFCGSGNVLLKYGTRDLNVVCGMLKIMPFTAVLFGGGALALALAGMPPFNI
E. coli3    LGLVGGLYHLLNHSLFKSVLFLGAGSVWFRTGHRDIEKLGGIGKKMPVISIAML--VGLMAMAALPPLNG
T. ther     EGAMGGLYLLAASGVYTGGLFLLAGRLYERTGTLEIGRYRGLAQSAPGLAALAL--ILFLAMVGLPGLSG
B. tau      WSYMGATALMIAHGLTSSMLFCLANSNYERIHSRTMILARGLQTLLPLMATWWL--LASLTNLALPPTIN
O. aries    WSYMGATALMIAHGLTSSMLFCLANSNYERVHSRTMILARGLQTLLPLMAAWWL--LASLTNLALPPSIN

                430       440       450       460       470       480       490
            ....|....|....|....|....|....|....|....|....|....|....|....|....|....|
S. mul      FVSEFLIFKAAAIGEQY----------LLMGIFAVALAIIFIADFSHFFLASFGKVEGEVVHNSEMKFSE
E. coli4    FLSEFMTITAG-LARNH----------LLIIVLLLLLLTLVLAGLVRMAARVLMAKPPQAVNRGDLGWLT
E. coli3    FAGEWVIYQSFFKLSNSGAFVARLLGPLLAVGLAITGALAVMCMAKVYGVTFLGAPRTKEAENATCAPLL
T. ther     FPGEFLTLLGA-YKASP----------WLAALAFLSVIASAAYALTAFQKTFWEEGGSGVKDLAGAEWGF
B. tau      LIGELFVVMST-FSWSN----------ITIILMGVNMVITALYSLYMLIMTQRGK-YTYHINNISPSFTR
O. aries    LIGELFVVMST-FSWSN----------ITIILMGLNMVITALYSLYMLITTQRGK-HTHHINNILPSFTR

                500       510       520       530       540       550       560
            ....|....|....|....|....|....|....|....|....|....|....|....|....|....|
S. mul      NFPLIALAILIVAFGIWQFDSFTFLLDESVKSIMKK----------------------------------
E. coli4    TSPMVILLVMMLAMGTHIPQPVIRILAGASTIVLSGTHDLPAQRSTWHDFLPSGTASVSEKHSER-----
E. coli3    MSVSVVALAICCVIGGVAAPWLLPMLSAAVPLPLEPANTTVSQPMITLLLIACPLLPFIIMAICKGDRLP
T. ther     ALLSVLALLLMGVFPGYFARGLHPLAEAFAKLLGGGA---------------------------------
B. tau      ENALMSLHILPLLLLTLNPKIILGPLY-------------------------------------------
O. aries    ENALMSLHMLPLLLLSLNPKIILGPLY-------------------------------------------

                570       580       590       600       610       620       630
            ....|....|....|....|....|....|....|....|....|....|....|....|....|....|
S. mul      ----------------------------------------------------------------------
E. coli4    ----------------------------------------------------------------------
E. coli3    SRSRGAAWVCGYDHEKSMVITAHGFAMPVKQAFAPVLKLRKWLNPVSLVPGWQCEGSALLFRRMALVELA
T. ther     ----------------------------------------------------------------------
B. tau      ----------------------------------------------------------------------
O. aries    ----------------------------------------------------------------------

                640
            ....|....|.
S. mul      -----------
E. coli4    -----------
E. coli3    VLVVIIVSRGA
T. ther     -----------
B. tau      -----------
O. aries    -----------
```

Supplementary Figure 17: Amino acid sequence alignment of HyfF/Nqo13/ND4. *S. mul* - *S. multivorans* AHJ13633.1 Hyf-like hydrogenase, membrane subunit F, *E. coli*4 - *E. coli* CTT40508.1 Hyf hydrogenase, membrane subunit F, *E. coli*3 - *E. coli* CAA35548.1 Hyc hydrogenase, membrane subunit 3, *T. ther* - *Thermus thermophilus* Q56228.2 NADH-quinone oxidoreductase subunit 13 (Nqo13), *B. tau* - *Bos taurus* P03910.1 NADH-ubiquinone oxidoreductase chain 4 (ND4), *O. aries* - *Ovis aries* O78755.1 NADH-ubiquinone oxidoreductase chain 4 (ND4). Amino acid color code: green - hydrophobic, red - charged, blue - polar/uncharged. Key charged residues are highlighted in yellow.

```
                   10        20        30        40        50        60        70
           ....|....|....|....|....|....|....|....|....|....|....|....|....|....|
S. mul     -----------MDFFYLLLQLISAILVAPLFDGISRKLRAKFQSRVGP------SIFQTYRDLLKLLKRG
E. coli4   ---MRQTLCDGYLVIFALAQAVILLMLTPLFTGISRQIRARMHSRRGP------GIWQDYRDIHKLFKRQ
E. coli3   -----------MSVLYPLIQALVLFAVAPLLSGITRVARARLHNRRGP------GVLQEYRDIIKLLGRQ
T. ther    MTWSYPVDPYWMVALKALLVVVGLLTAFAFMTLIERRLLARFQVRMGPNRVGPFGLLQPLADAIKSIFKE
B. tau     -----------MFMINILMLIIPILLAVAFLTLVERKVLGYMQLRKGPNVVGPYGLLQPIADAIKLFIKE
O. aries   -----------MFMINVLTLIIPILLAVAFLTLVERKVLGYMQFRKGPNVVGPYGLLQPIADAIKLFIKE

                   80        90       100       110       120       130       140
           ....|....|....|....|....|....|....|....|....|....|....|....|....|....|
S. mul     RTKSHSTS-YIYQIAPYVLFVSAAAMFCALPIAYDIR-----SSVLSQFSDIFVLLYLGALFRFMFIIAG
E. coli4   EVAPTSSG-LMFRLMPWVLISSMLVLAMALPLFITV-------SPFAGGGDLITLIYLLALFRFFFALSG
E. coli3   SVGPDASG-WVFRLTPYVMVGVMLTIATALPVVTVG-------SPLPQLGDLITLLYLFAIARFFFAISG
T. ther    DIVVAQADRFLFVLAPLISVVFALLAFGLIPFGPPGSFFGYQPWVINLDLGILYLFAVSELAVYGIFLSG
B. tau     PLRPATSSASMFILAPIMALGLALTMWIPLPMPYP---------LINMNLGVLFMLAMSSLAVYSILWSG
O. aries   PLRPATSSISMFILAPILALTLALTMWIPLPMPYP---------LINMNLGVLFMLAMSSLAVYSILWSG

                  150       160       170       180       190       200       210
           ....|....|....|....|....|....|....|....|....|....|....|....|....|....|
S. mul     IDTANPFAGVSASREGTLGFYTEEIAVICLVVVMMGTGSTNLAYITNLVQEGQYGYAFPSFSIAATAFLW
E. coli4   LDTGSPFAGVGASRELTLGILVEPMLILSLLVLALIAGSTHIEMISNTLAMG-WNSPLT-TVLALLACGF
E. coli3   LDTGSPFTAIGASREAMLGVLVEPMLLLGLWVAAQVAGSTNISNITDTVYHWPLSQSIP-LVLALCACAF
T. ther    WASGSKYSLLGSLRSSASLISYELGLGLALLAPVLLVGSLNLNDIVNWQKEHGWLFLYA--FPAFLVYLI
B. tau     WASNSKYALIGALRAVAQTISYEVTLAIILLSVLLMSGSFTLSTLITTQ-EQMWLILPA--WPLAMMWFI
O. aries   WASNSKYALIGALRAVAQTISYEVTLAIILLSVLLMNGSFTLSTLIITQ-EQVWLIFPA--WPLAMMWFI

                  220       230       240       250       260       270       280
           ....|....|....|....|....|....|....|....|....|....|....|....|....|....|
S. mul     VMYVETGRKPYDLAEAEQELQEGVLGEYCGKDLAIIDVAILLKQFTMLGFFLVIFIPW------SFENPI
E. coli4   ACFIEMGKIPFDVAEAEQELQEGPLTEYSGAGLALAKWGLGLKQVVMASLFVALFLPFGRAQELSLACLL
E. coli3   ATFIEMGKLPFDLAEAEQELQEGPLSEYSGSGFGVMKWGISLKQLVVLQMFVGVFIPWGQMETFTAGGLL
T. ther    ASMAEAARTPFDLPEAEQELVGGYHTEYSSIKWALFQMAEYIHFITASALIPTLFLGGWTMPVLEVPYLW
B. tau     STLAETNRAPFDLTEGESELVSGFNVEYAAGPFALFFMAEYANIIMMNIFTAILFLGTSHNPHMPELYTI
O. aries   STLAETNRAPFDLTEGESELVSGFNVEYAAGPFALFFMAEYANIIMMNIFTTTLFLGAFHNPYMPELYTI

                  290       300       310       320       330       340       350
           ....|....|....|....|....|....|....|....|....|....|....|....|....|....|
S. mul     LSLIAFLAEVGFLYVMGVFIDNFGPRFTVNNGIKRTMLFALA--------------------ISCTSLLL
E. coli4   TSLVVTLLKVLLIFVLASIAENTLARGRFL-LIHHVTWLGFS--------------------LAALAWVF
E. coli3   LALVIAIVKLVVGVLVIALFENSMARLRLD-ITPRITWAGFG--------------------FAFLAFVS
T. ther    MF-LKIAFFLFFFIWIRATWFRLRYDQLLRFGWGFLFPLALLWFLVTALVVALDLPRTYLLYLSALSFLV
B. tau     NFTIKSLLLTMSFLWIRASYPRFRYDQLMHLLWKNFLPLTLA-------------------LCMWHVSLPI
O. aries   NFTIKSLLLSITFLWIRASYPRFRYDQLMHLLWKNFLPLTLA-------------------LCMWHVSLPI


                  360       370
           ....|....|....|...
S. mul     YIMGI-------------
E. coli4   WLTGL-------------
E. coli3   LLAA--------------
T. ther    LLGAVLYTPKPARKGGGA
B. tau     LTSGIPPQT---------
O. aries   LLSSIPPQT---------
```

Supplementary Figure 18: Amino acid sequence alignment of HyfC/Nqo8/ND1. *S. mul* - *S. multivorans* AHJ13631.1 Hyf-like hydrogenase, membrane subunit C, *E. coli*4 - *E. coli* AAB88565.1 Hyf hydrogenase, membrane subunit C, *E. coli*3 - *E. coli* CAA35549.1 Hyc hydrogenase, membrane subunit 4, *T. ther* - *Thermus thermophilus* Q60019.1 NADH-quinone oxidoreductase subunit 8 (Nqo8), *B. tau* - *Bos taurus* P03887.1 NADH-ubiquinone oxidoreductase chain 1 (ND1), *O. aries* - *Ovis aries* O78747.1 NADH-ubiquinone oxidoreductase chain 1 (ND1). Amino acid color code: green - hydrophobic, red - charged, blue - polar/uncharged. Key charged residues of the connection of the two half-channels are highlighted in yellow. Key charged residues from the first-half channel are highlighted in dark blue.

Supplementary Table 6: Distribution of the *hyf* operon among different Epsilonproteobacteria and possible formation of a formate dehydrogenase lyase complex. FHL-co - *hyf* operon associated with a formate dehydrogenase, Hyf-foc - *hyf* operon associated with a formate channel (*focB*). For an overview of the organization of different *hyf* operons see Supplementary Figure 1.

	FHL-co	Hyf-Foc	Hyf
***Sulfurospirillum** multivorans*			■
S. cavolei			■
S. arsenophilum			■
S. halorespirans[2]			■
S. arcachonense	■		■
Candidatus *S. diekertiae*[3]	■		■
S. sp. JPD-1	■		■
S. barnesii			■
S. deleyianum			■
S. cavolei MES[4]			■
S. sp. UCH001			■
S. sp. UBA11407			■
S. sp. UBA12182			■
S. sp. SCADC			■
S. sp. AM-N	■		
***Arcobacter** nitrofigilis*	■		
A. marinus	■		
A. molluscorum	■		
A. sp. F138-33	■		
***Campylobacter** concisus*		■	
C. curvus		■	
C. gracilis		■	
C. mucosalis		■	
C. pinnipediorum		■	
C. showae		■	
C. sp. FOBRC14		■	
C. sp. 10_1_50		■	
C. fetus			■
C. hyointestinalis			■
C. iguaniorum			■
C. sputorum			■
***Wolinella** succinogenes*			■
***Caminibacter** mediatlanticus*	■		
***Lebetimonas** spp.*	■		

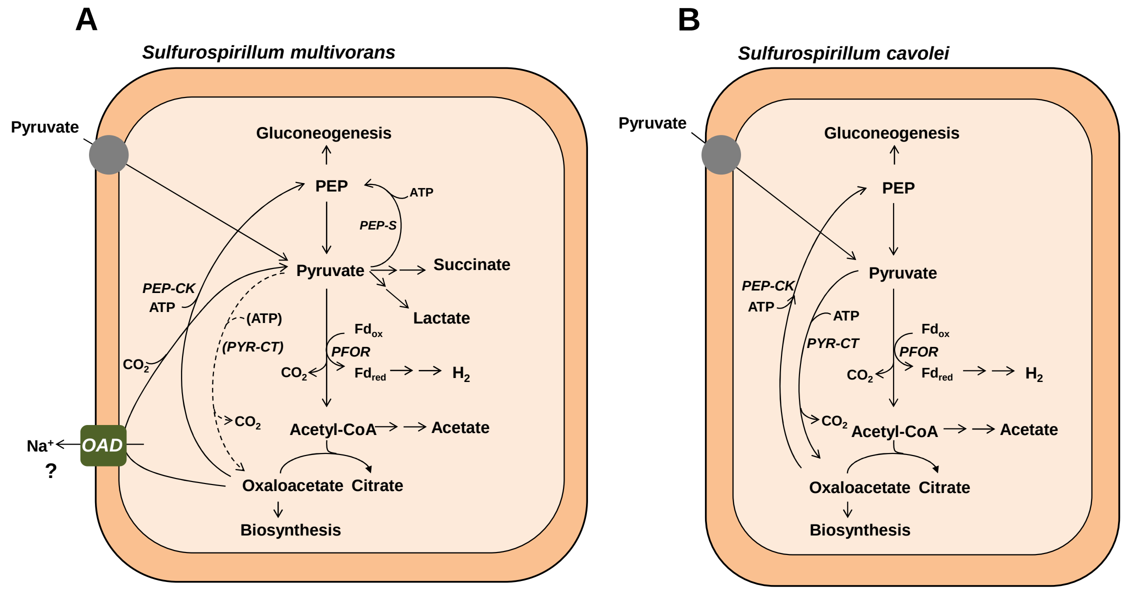

Supplementary Figure 19: Biosynthetic reactions including gluconeogenesis from pyruvate in (A) *Sulfurospirillum multivorans* and (B) *Sulfurospirillum cavolei*. Pyruvate carboxylation via pyruvate carboxylase in *S. cavolei* proceeds presumably in the reverse direction via oxaloacetate decarboxylase in *S. multivorans*, since an oxaloacetate decarboxylase subunit is encoded in *S. multivorans* (see also Supplementary Figure 8). PEP-S - PEP-synthetase, PEP-CK - PEP-carboxykinase, PYR-CT - pyruvate carboxylase, OAD - oxaloacetate decarboxylase, PFOR - pyruvate:ferredoxin oxidoreductase, Fd - ferredoxin.

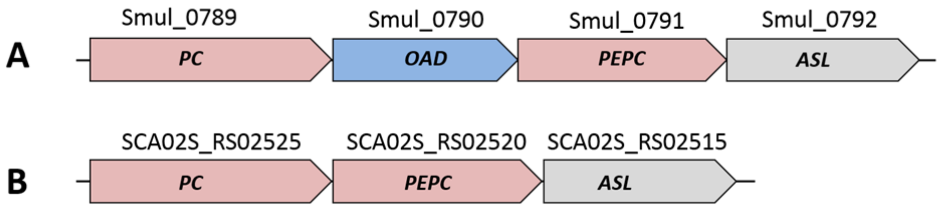

Supplementary Figure 20: Cluster of genes related to pyruvate metabolism in (A) *S. multivorans and (B) *S. cavolei**.** Locus tags of genes are given above the genes. PC - pyruvate carboxylase, OAD - oxaloacetate decarboxylase, PEPC - phosphoenolpyruvate carboxykinase ASL, - argininosuccinate lyase. A*: Identified in *S. deleyianum*, *S. halorespirans*, S. sp. SCADC, *S. barnesii*, S. sp. UBA12182, S. sp. UBA11407, *S. arcachonense*, S. sp. AM-N. B*: Identified in S. UCH001, *S. cavolei* strains MES and UCH003, *S. arsenophilum*, S. sp. JPD-1.

SUPPLEMENTARY NOTE 2

Determination of lactate dehydrogenase activity in crude extracts of *S. multivorans*

Lactate-producing and lactate-oxidizing enzyme activity was measured anoxically in quartz cuvettes using a Cary 100 spectrophotometer (Agilent Technologies, Waldbronn, Germany) with NADH and NAD^+ as electron donor/acceptor. Lactate-producing activity was followed by the decrease of absorbance at 340 nm of NADH. The assay mixture contained: 50 mM Tris-HCl (pH 7.5), 0.2 mM NADH, 1 mM KCN and 30 mM sodium pyruvate (pH 7.5). The reaction was started by the addition of crude extracts to 1 ml of the reaction mixture and the assay was incubated at room temperature. Lactate oxidation activity with NAD^+ was monitored by the increase of absorbance at 340 nm in a hydrazine-containing buffer; hydrazine was applied to trap pyruvate and thus shift the equilibrium of the normally thermodynamically unfavorable LDH-catalyzed oxidation of lactate with NAD^+ towards pyruvate formation. In addition, an excess of NAD^+ was applied to aid in overcoming the thermodynamic barrier. The reaction mixture contained 50 mM Tris-HCl (pH 9.0), 0.5 mM NAD^+, 1 mM KCN, 30 mM sodium pyruvate (pH 7.5) and 30 mM hydrazine. Commercially available lactate dehydrogenase (~95% purity, 1.100 U/mg, Sigma Aldrich, Steinheim, Germany) served as positive control. No NAD^+- or NADH-dependent enzyme activity was measured in crude extracts.

Additionally, artificial electron donors such as sodium dithionite, methyl viologen and duroquinone (2,3,5,6-Tetramethyl-1,4-benzoquinone) were tested for enzyme-mediated lactate formation from pyruvate. NADH was replaced by 20 mM sodium dithionite, 10 mM reduced (with sodium dithionite) methyl viologen (98% purity, Sigma Aldrich, Steinheim, Germany) or 10 mM duroquinol (midpoint redox potential E°' = -240 mV) (97% purity, Sigma Aldrich, Steinheim, Germany). Lactate was measured by HPLC (AMINEX HPX-87H column, 7.8x300 mm, BioRad, Munich, Germany).

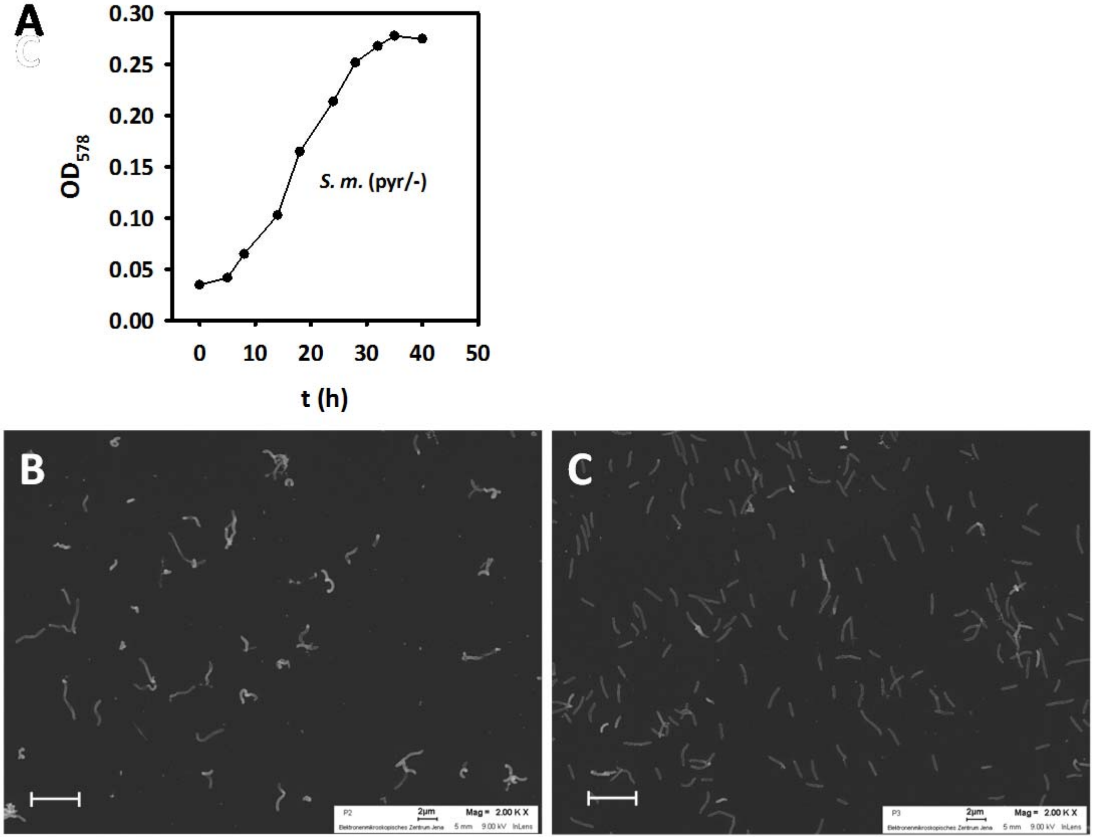

Supplementary Figure 21: Cultivation and field emission scanning electron microscopic (FE-SEM) analysis. (A) Pyruvate fermentation of *S. multivorans* pure culture. Electron micrographs of *S. multivorans* growing fermentatively in modified *Methanococcus* basal medium on pyruvate (B) and lactate/fumarate (C). For a detailed medium description see Methods. S. m. - *S. multivorans*. Scale bars correspond to 5 µm in B and C.

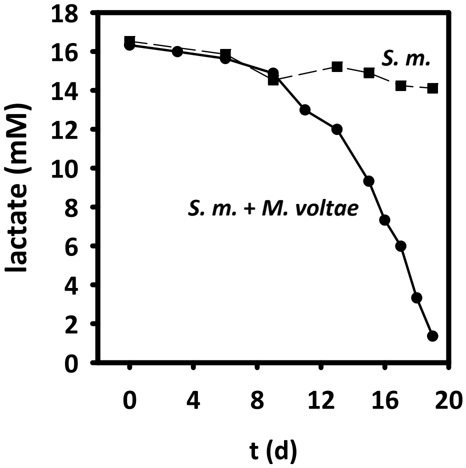

Supplementary Figure 22: Lactate consumption of an *S. multivorans/M. voltae* co-culture with *S. multivorans* cells not adapted to fermentation. *S. multivorans* pure culture was used as control. S. m. - *S. multivorans,* M. voltae - *Methanococcus voltae*.

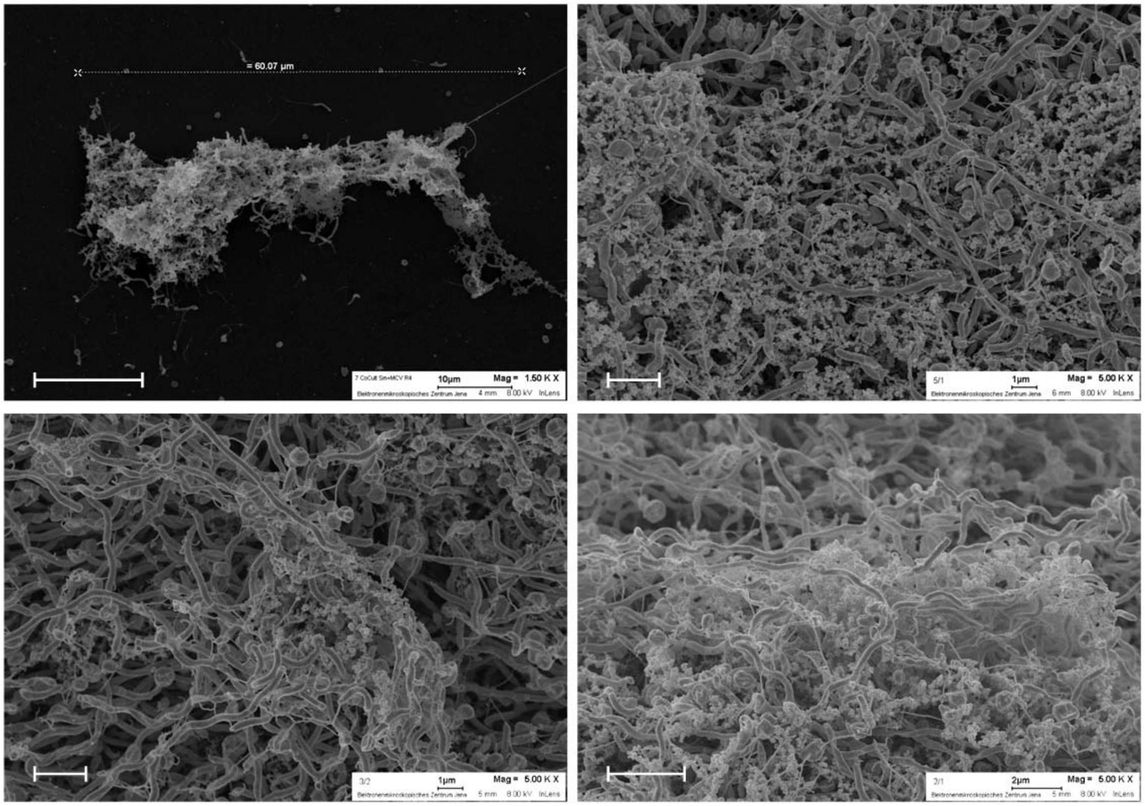

Supplementary Figure 23: Syntrophic co-culture of *S. multivorans* and *M. voltae*. Field emission scanning electron microscopic (FE-SEM) analysis of the formed aggregates. Different areas and magnifications are depicted in the images. Scale bars correspond to 200 µm (upper left), 3 µm (upper right), 2 µm (lower left) and 2 µm (lower right).

References

1. Marreiros, B.C., Batista, A.P., Duarte, A.M. & Pereira, M.M. A missing link between complex I and group 4 membrane-bound [NiFe] hydrogenases. Biochim Biophys Acta 1827, 198-209 (2013).

2. Goris, T. et al. The complete genome of the tetrachloroethene-respiring Epsilonproteobacterium *Sulfurospirillum halorespirans*. J Biotechnol 255, 33-36 (2017).

3. Ross, D.E., Marshall, C.W., May, H.D. & Norman, R.S. Comparative Genomic Analysis of *Sulfurospirillum cavolei* MES Reconstructed from the Metagenome of an Electrosynthetic Microbiome. PLoS One 11, e0151214 (2016).

4. Buttet G.F., Murray A.M., Goris T., Burion M., Jin B., Rolle M., Holliger C. & Maillard J. Coexistence of two distinct *Sulfurospirillum* populations respiring tetrachloroethene-genomic and kinetic considerations. FEMS Microbiol Ecol 94, fiy018 (2018).
