# Supplementary material for: Hydrogen production by Sulfurospirillum species enables syntrophic interactions of Epsilonproteobacteria
Source: Nat Commun. 2018 Nov 19;9:4872. doi: 10.1038/s41467-018-07342-3 (PMC6242987; doi:10.1038/s41467-018-07342-3)
Supplement: Supplementary file 3 — Description of Additional Supplementary Files [file 41467_2018_7342_MOESM3_ESM.pdf]

Description of Additional Supplementary Files

File Name: Supplementary Data 1
Description: This dataset contains unprocessed area values and interim values of the statistical analyses of <i>S. multivorans</i> and <i>S. cavolei</i> on pyruvate alone vs. pyruvate/fumarate.

File Name: Supplementary Data 2
Description: This dataset contains proteome data of <i>S. multivorans</i> and <i>S. cavolei</i> pyruvate fermentation vs. pyruvate oxidation/fumarate respiration

File Name: Supplementary Data 3
Description: This dataset contains unprocessed area values and interim values of the statistical analyses of <i>S. multivorans</i> pyruvate fermentation adaptation and lactate oxidation proteomes.

File Name: Supplementary Data 4
Description: This dataset contains proteome data of <i>S. multivorans</i> grown with lactate/fumarate vs. pyruvate/fumarate.

File Name: Supplementary Data 5
Description: This dataset contains proteome data of <i>S. multivorans</i> pyruvate fermentation-adaptated cells vs. non-adapted cells.
